# CHANGES IN LIVING ARRANGEMENT PREFERENCE AMONG OLDER TAIWANESE: A MULTILEVEL AGE-PERIOD COHORT ANALYSIS

**DOI:** 10.1093/geroni/igad104.0185

**Published:** 2023-12-21

## Abstract

Coresidence between older parents and adult children has been a traditional form of living arrangements in East Asia. The majority of older Taiwanese parents still shares the same residence with their adult children. However, recent research shows two contrasting trends: older persons tend to prefer independent living, while younger adults still express their filial responsibilities. Does the difference indicate changes in living arrangement preferences by successive cohort, or does it suggest an age pattern of coresidence preference among people who were growing old? Data from the Taiwan Longitudinal Study on Aging (TLSA) were used to compile an analytic sample aged 50 and older in 1996 with six follow-up interviews to 2019. Four birth cohorts between 1916 and 1953 were identified. The multilevel hierarchical age-period-cohort model (HAPC) were used to examine the effects of age, period, and cohort on potential changes in coresidence preference. Preliminary results suggested that coresidence preference among the post-war birth cohort increased with age, while no clear age patterns were found among other older cohorts. After entering older ages, all birth cohorts converged toward the similar coresidence preference. Findings suggest living with adult children may still be the primary form of living arrangements for older Taiwanese to ensure their sources of care. Despite that realization of coresidence preference would be crucial for older persons’ well-being, understanding how coresidence preference changes over time during one’s life course can further help us envision the future household structure.

